# Pan-cancer analysis of intratumor heterogeneity associated with patient prognosis using multidimensional measures

**DOI:** 10.18632/oncotarget.26485

**Published:** 2018-12-28

**Authors:** Chie Kikutake, Minako Yoshihara, Tetsuya Sato, Daisuke Saito, Mikita Suyama

**Affiliations:** ^1^ Medical Institute of Bioregulation, Kyushu University, Fukuoka 812-8582, Japan

**Keywords:** variant allele frequency (VAF), The Cancer Genome Atlas (TCGA), next-generation sequencing, intratumor heterogeneity, prognosis

## Abstract

Human cancers accumulate various mutations during development and consist of highly heterogeneous cell populations. This phenomenon is called intratumor heterogeneity (ITH). ITH is known to be involved in tumor growth, progression, invasion, and metastasis, presenting obstacles to accurate diagnoses and effective treatments. Numerous studies have explored the dynamics of ITH, including constructions of phylogenetic trees in cancer samples using multiregional ultradeep sequencing and simulations of evolution using statistical models. Although ITH is associated with prognosis, it is still challenging to use the characteristics of ITH as prognostic factors because of difficulties in quantifying ITH precisely. In this study, we analyzed the relationship between patient prognosis and the distribution of variant allele frequencies (VAFs) in cancer samples (*n* = 6,064) across 16 cancer types registered in The Cancer Genome Atlas. To measure VAF distributions multidimensionally, we adopted parameters that define the shape of VAF distributions and evaluated the relationships between these parameters and prognosis. In seven cancer types, we found significant relationships between prognosis and VAF distributions. Moreover, we observed that samples with a larger amount of mutations were not necessarily linked to worse prognosis. By evaluating the ITH from multidimensional viewpoints, it will be possible to provide a more accurate prediction of cancer prognosis.

## INTRODUCTION

Cancer is indicated via dysregulated cell growth, proliferation, and cell cycle progression. Cancer cells often consist of heterogeneous populations with various mutations rather than composed of homogeneous populations [1–3]. Previous studies demonstrated that cancer develops from mutations in certain driver genes and eventually accumulates various genetic mutations through cell growth, leading to intratumor heterogeneity (ITH) [4]. ITH may be associated with drug resistance and disease recurrence [5].

The recent advent of next-generation sequencing technologies allows us to analyze the process of accumulated mutations in cancer cells during progression. Using multiregional sequencing, we can construct the “cancer evolutionary tree,” which depicts clonal and subclonal mutations as the trunk and branches [4, 6, 7]. Another approach is using variant allele frequencies (VAFs). VAFs are able to estimate the fraction of tumor populations containing mutations in cancer cells [8, 9]. These studies have revealed that cancer evolution is highly diverse and complex among cancer types and individuals.

Despite these efforts, the relationship between the heterogeneity in cancer cells and clinical outcomes of patients remains insufficiently understood. Several measures have been developed and used for the analysis. For example, some algorithms estimate the number of subclonal populations and quantify the extent of ITH [10, 11]. A previous study suggested a nonlinear relationship between the extent of ITH and prognosis [12]. Moreover, mutant-allele tumor heterogeneity (MATH) [13] scores represent the variance of VAFs, and the entropy-based mutation allele fraction (EMAF) [14] represents uncertainty of somatic mutation patterns. It is reported that higher scores of these measures were significantly associated with poorer prognosis in head and neck squamous cell carcinoma and in non-small cell lung cancer, respectively. These measures are, however, not robust for all cancer types, mainly because the one-dimensional measure is insufficient for expressing the complexity of cancer evolution. Furthermore, it is difficult to one-dimensionally infer the status of cancer cells in the evolutionary process.

**Figure 1:**
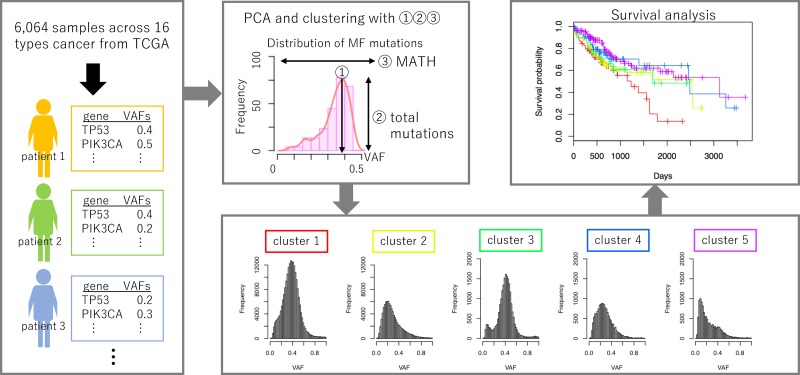
Graphical summary of the workflow VAFs are calculated for each mutation in patient samples obtained from TCGA. Using three parameters derived from the shape of the VAF distribution, samples are classified into five clusters. Survival analysis was performed to compare the prognoses among samples belonging to each cluster.

In this study, to evaluate the multidimensional nature of cancer heterogeneity, we adopted three parameters that define the shape of VAF distribution, such as the number of mutations, peak position, and variance. We used VAFs from 6,064 The Cancer Genome Atlas (TCGA) samples of 16 cancer types. Using the three parameters, we analyzed the association between the shape of the VAF distribution and the prognosis of each cancer type (Figure 1).

## RESULTS

### Clustering all cancer samples using VAF distribution parameters

We obtained somatic mutation data for each sample (*n* = 6,064) across 16 cancer types from the TCGA repository: bladder urothelial carcinoma (BLCA), breast adenocarcinoma (BRCA), cervical squamous cell carcinoma and endocervical adenocarcinoma (CESC), colon adenocarcinoma (COAD), glioblastoma multiforme (GBM), head and neck squamous cell carcinoma (HNSC), kidney renal clear cell carcinoma (KIRC), lower-grade glioma (LGG), liver hepatocellular carcinoma (LIHC), lung adenocarcinoma (LUAD), lung squamous cell carcinoma (LUSC), ovarian serous carcinoma (OV), prostate adenocarcinoma (PRAD), skin cutaneous melanoma (SKCM), thyroid carcinoma (THCA), and uterine corpus endometrial carcinoma (UCEC). The total more functional (MF) mutations, which are mutations classified as “probably damaging” or “possibly damaging” by PolyPhen-2, were 469,553 (Supplementary Table 1). We used three parameters that define the shape of VAF distributions of MF mutations in each sample: the corresponding VAF with the maximum value for probability density function of VAF distribution of MF mutations (m_Peak), log2[the total number of MF mutations] (m_Count) and MATH score for MF (m_MATH). First, we calculated the correlation coefficients for all possible combinations of the three parameters to examine whether they are independent variables. Since correlation coefficients were −0.44, 0.03, and 0.00, which were observed between m_Peaks vs. m_MATH, m_Peak vs. m_Count, and m_Count vs. m_MATH, respectively, we considered the parameters could be used as independent variables representing the characteristics of VAF distributions.

**Figure 2:**
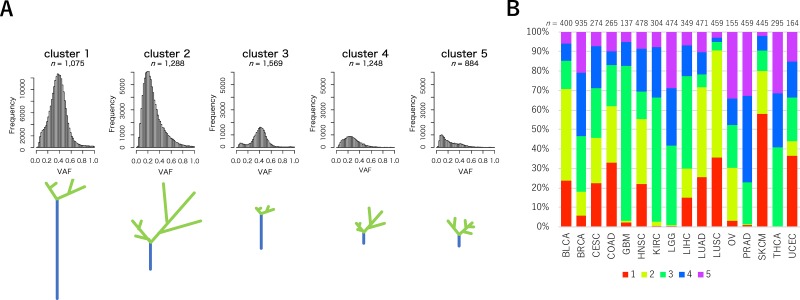
VAF distributions of the five clusters and sample frequencies among 16 cancer types **(A)** Histograms for VAFs of MF mutations of the five clusters. The horizontal axis indicates VAF values and the vertical axis indicates mutation frequencies. Trunk-branch models are shown at the bottoms of the histograms. The number of mutations with higher VAFs are represented as the trunk of the tree (blue), whereas the number of mutations with lower VAFs are represented as the branches (green). **(B)** The proportions of the five clusters for each cancer type. The number of tumor samples represent analyzed cases. Color codes for the five clusters are indicated at the bottom of the bar plots.

We next examined the VAF distribution patterns of individual samples across 16 cancer types. Principal component analysis (PCA) was performed using the three parameters defining the shape of VAF distributions in 6,064 samples, and we extracted two principal components. Each principal component (PC1 and PC2) accounted for 48.2% and 33.3% of the total variance in the data, respectively (Supplementary Figure 1A). Using the two principal components, a total of 6,064 samples were divided into five clusters by the k-medoids algorithm (Supplementary Figure 1B). We used k = 5 for clustering because it was possible to obtain distinct VAF distribution by dividing samples into five clusters rather than k = 3, 4, or 6. We drew histograms of VAFs assembled from all mutations in samples belonging to each cluster and created trunk-branch models of mutations in tumors (Figure 2A). For each type of cancer, we calculated the proportions of the five clusters (Figure 2B and Supplementary Table 2). We also calculated the median values of the three parameters for the five clusters (Table 1).

**Table 1:** Parameter characteristics regarding VAF distribution

Parameters	Cluster				
	1	2	3	4	5
median of m_Peak	0.376	0.229	0.424	0.271	0.140
median of m_Counts	6.794	6.119	4.000	3.807	3.807
median of m_MATH	0.186	0.320	0.133	0.251	0.473

As a result, the samples in clusters 1 and 2 harbored more MF mutations than the other three clusters. Since the VAF distributions showed that samples in cluster 1 had more MF mutations with higher VAF than lower VAF, while the samples in cluster 2 had more MF mutations with lower VAF than higher VAF, they were predicted to have accumulated clonal mutations in cluster 1 and subclonal mutations in cluster 2, respectively [15, 16]. As shown in Figure 2B, the frequencies of samples in cluster 1 were relatively higher in SKCM and LUSC. This observation was consistent with a recent study by McGranahan and colleagues, which indicated that, in some cancer types, including melanoma and lung cancer, mutations accumulated prior to carcinogenesis [6]. The frequencies in cluster 2 were also higher in BLCA, LUAD, and LUSC. In these cancers, a large subclonal mutation burden was previously observed [6].

Samples in clusters 3, 4, and 5 had fewer MF mutations than clusters 1 and 2. The frequencies of these clusters were relatively higher in GBM, KIRC, LGG, PRAD, and THCA. Previous studies showed that kidney, brain, and thyroid tumors had a relatively lower number of mutations [17]. We next focused on the differences among the three clusters. Samples in cluster 3 had higher m_Peaks and lower m_MATH, whereas samples in cluster 5 had lower m_Peak and higher m_MATH (Table 1). This trend can be interpreted as MF mutations occurring in the early stages of cancer development and maintained through cancer progression, without further accumulating a large number of MF mutations among samples in cluster 3. In cluster 4, expansion of some subclones with certain MF mutations might occur during cancer progression under strong positive selection [18, 19]. In contrast, samples in cluster 5 had MF mutations that possibly occurred under neutral cancer evolution [20]. The frequencies of clusters 4 and 5 were especially higher in PRAD. The results described above were partly supported by the “Evolutionary Trees” illustrated in a previous study [6].

### Genetic characteristics of five clusters

To evaluate the clusters’ genetic characteristics, we calculated MF mutation frequencies of each gene for 16 cancer types and examined the 10 genes with the highest frequency of mutations in each cluster (Supplementary Figure 2). In BRCA, the frequencies of MF mutation in *PIK3CA* in clusters 3, 4, and 5 (35.5%, 40.5%, and 32.5%, respectively), in which the m_Count was small, were relatively high. Once mutations in *PIK3CA* occurred, without a striking increase in the number of other mutations, cells may remain genetically stable. The MF mutation frequency of *CTNNB1* (20.0%) in cluster 3 in LIHC was also high. This result suggests that liver cancer cells with mutations in the driver gene *CTNNB1*, which have been generated in the earlier stage of cancer development, occupied in the cancer cell population. Although high frequency MF mutations (in SKCM and UCEC) were mainly found in cluster 1, the samples in cluster 2, which also had a large number of MF mutations, had mutations with lower frequencies. This result showed that there are common mutated genes in cluster 1.

**Figure 3:**
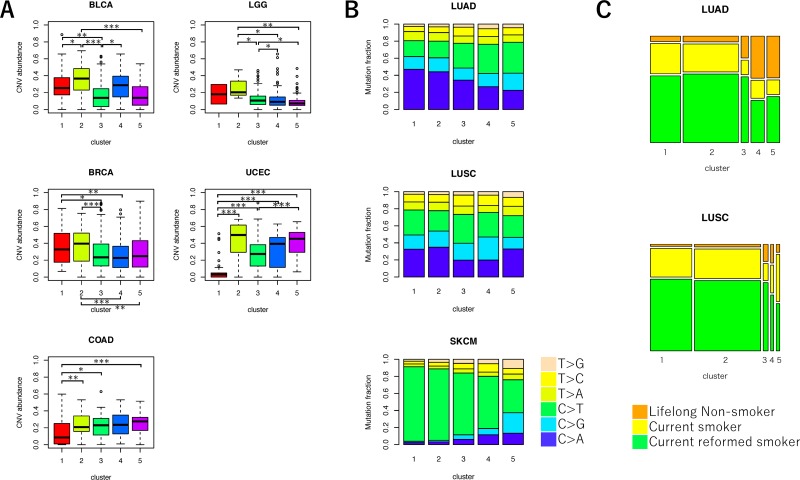
Comparison of genetic characteristics among the five clusters of VAF distributions **(A)** Boxplot for CNV abundance of samples in each cluster. ANOVA, followed by Tukey's honest significant difference test, was performed. ^*^P < 0.05, ^**^P < 0.01, and ^***^P < 0.001. **(B)** Bar plot for the frequencies of mutation spectra in three cancer types. The fractions of six mutation types in each cluster were shown. **(C)** Mosaic plot for the frequencies of patients’ smoking history in LUAD and LUSC. Color codes for smoking history are indicated at the bottoms of the plots.

Moreover, we evaluated the extent of ITH via genomic instability. A previous study showed that genomic instability is correlated with ITH [12]. We assessed the association between copy number variant (CNV) abundance as the extent of the genomic instability and five clusters for each cancer type [21]. The tendencies of CNV abundance for the five clusters varied widely among cancer types (Figure 3A, Supplementary Figure 3, and Supplementary Table 2). In our study, the samples in cluster 2 was predicted to have the highest ITH level due to a large number of mutations with lower VAF. Although we hypothesized the samples in cluster 2 would have the highest CNV levels, only BLCA, BRCA, and LGG had the highest levels of CNV abundance.

We also examined the MF mutation spectrum between the five clusters (Figure 3B and Supplementary Figure 4). In melanoma, the frequency of C>T transitions decreases, and the frequency of T>G transversions increases among branch mutations compared to trunk mutations [22, 23]. The frequency of C>T mutations was significantly lower among samples in clusters 2, 3, 4, and 5 than samples in cluster 1 (cluster 2: false discovery rate (FDR)-adjusted *P* < 0.001, cluster 3: FDR-adjusted *P* < 0.001, cluster 4: FDR-adjusted *P* < 0.001, and cluster 5: FDR-adjusted *P* < 0.001), which was predicted to have a larger number of trunk mutations in SKCM. Therefore, most mutations in samples with fewer mutations were proposed to occur in later, rather than earlier, stages of cancer development [2, 24]. The frequency of C>A transversions decreased among branch mutations compared to trunk mutations in LUAD and LUSC samples [25]. We observed that the frequency of C>A decreased in LUAD samples in clusters 2, 3, 4, and 5 compared with samples in cluster 1 (cluster 2: FDR-adjusted *P* < 0.001, cluster 3: FDR-adjusted *P* < 0.001, cluster 4: FDR-adjusted *P* < 0.001, and cluster 5: FDR-adjusted *P* < 0.001). We also observed that the frequency of C>A decreased in LUSC samples in clusters 3 and 4 compared with samples in cluster 1 (cluster 3: FDR-adjusted *P* < 0.001 and cluster 4: FDR-adjusted *P* < 0.001). As a result, samples in these clusters may include later mutations compared to earlier mutations. We also found that the observed mutation spectrum was strongly associated with smoking history in LUAD [25, 26]. The proportion of “never smoker” samples were significantly higher in clusters 3, 4, and 5 than in cluster 1 (cluster 3: FDR adjusted *P* = 0.027, cluster 4: FDR adjusted *P* < 0.001, and cluster 3: FDR adjusted *P* < 0.001) (Figure 3C).

### Clinical characteristics of samples in the five clusters of VAF distributions

**Figure 4:**
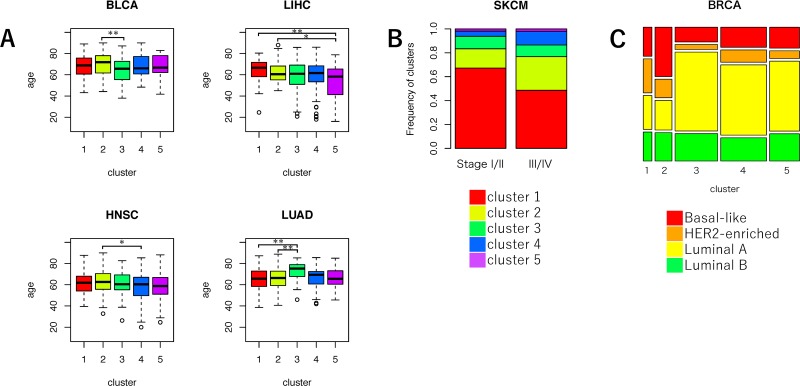
Comparison of clinical characteristics among the five clusters of VAF distributions **(A)** Boxplot for the average age in each cluster. ANOVA, followed by Tukey's honest significant difference test, was performed. ^*^P < 0.05, ^**^P < 0.01, and ^***^P < 0.001. **(B)** Bar plot for the frequencies of five clusters in lower (I/II) or higher cancer stage (III/IV) samples in SKCM. Color codes for the five clusters are indicated at the bottom of the bar plots. **(C)** Mosaic plot of the frequencies of BRCA molecular subtypes. Color codes for the molecular subtypes are indicated at the bottom of the plots.

To investigate the clinical characteristics of samples in the five clusters of VAF distributions, we examined the distributions of age at cancer diagnosis, tumor stage, gender, and breast cancer subtypes (Supplementary Table 2). We found that there were significant differences in the mean age among samples in the five clusters of the four cancer types (BLCA: FDR-adjusted *P* = 0.041, HNSC: FDR-adjusted *P* = 0.041, LIHC: FDR-adjusted *P* = 0.041, and LUAD: FDR-adjusted *P* = 0.041) (Figure 4A). For three of the cancer types (except LUAD), the average age of samples in either cluster 1 or 2 was relatively higher compared to other clusters. As mentioned above, samples in clusters 1 and 2 supposedly accumulated a large number of MF mutations during cancer development. This result was consistent with the fact that mutation frequencies increase with the patient's age [27, 28].

Significant differences in the proportion of the five clusters between tumor stages I/II and III/IV were observed only in SKCM (FDR-adjusted *P* = 0.009) (Figure 4B). In SKCM, the frequency of samples in cluster 1 was higher in lower tumor stages than in higher tumor stages. The frequency of cluster 1 significantly decreased, and the frequencies of clusters 2 and 4 significantly increased in the higher stage group (cluster 1: FDR-adjusted *P* = 0.001, cluster 2: FDR-adjusted *P* = 0.024, and cluster 4: FDR-adjusted *P* = 0.042). This result suggested that melanoma's aggressiveness may increase due to subclonal mutations that occur in later stages of cancer evolution.

In contrast, significant differences in the proportion of the five clusters between the gender were not observed in any types of cancer, indicating no relationships between gender and VAF distribution.

To evaluate the relationship between breast cancer subtypes and the five clusters, we calculated the frequencies of four major breast cancer subtypes (Basal-like, HER2-enriched, Luminal A, and Luminal B) in each cluster (Figure 4C) [29]. As a result, the frequency of basal-like breast cancer, which is a subtype included in triple negative breast cancer, was the highest in cluster 2 (34.8%). Furthermore, the frequency of HER2-enriched breast cancer was the highest in cluster 1 (25.9%). These results indicated that the evolutionary process of breast cancer may be very different depending on subtype [30].

### Relationship between samples in the five clusters of VAF distributions and prognosis

To assess the relationship between samples in each cluster of VAF distributions and their clinical outcomes, we separately constructed univariate Cox models for each cancer type. In this analysis, cluster 2 was used as a reference for each cancer type, because they are predicted to have the highest ITH level. As a result, significant association between samples in cluster 2 and survival was evident in only two cancer types (LUSC and SKCM). Compared to samples in some other clusters, samples in cluster 2 were associated with better prognosis in LUSC and SKCM (vs. cluster 4 in LUSC, *P* < 0.001; vs. cluster 1 in SKCM, *P* = 0.021; vs. cluster 3 in SKCM, *P* < 0.001; and vs. cluster 4 in SKCM, *P* = 0.001) (Supplementary Table 3). This result suggested that the relationship between ITH and prognosis is not uniform and may be different for cancer types.

**Figure 5:**
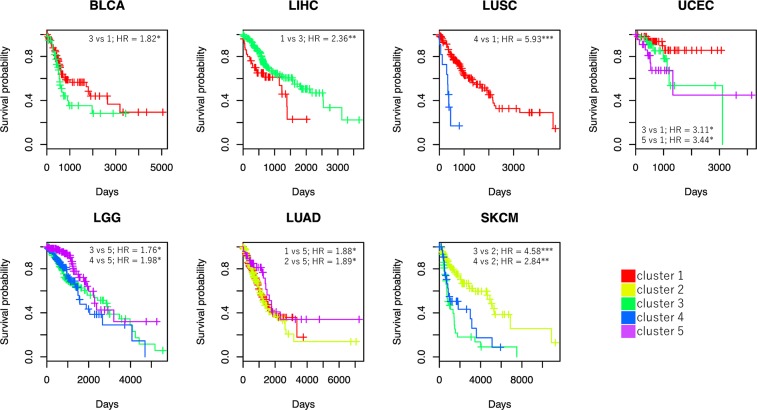
Survival curves and HRs derived from Cox proportional hazards regression Survival curves are shown for clusters in seven cancer types with significant HRs. Cox analysis adjusted for other covariates was performed using clusters with sample sizes ≥ 10. For each cancer type, one of the five clusters was selected as a reference so that all HRs were calculated as more than 1 (e.g., in the case of BLCA, cluster 1 was used as a reference). The horizontal axis indicates time (days), and the vertical axis shows survival probability. ^*^P < 0.05, ^**^P < 0.01, and ^***^P < 0.001.

To examine the relationship between samples in each cluster of VAF distributions and their clinical outcomes in detail, we took covariates into consideration. The aim of this analysis was to evaluate the differences in the states of tumor cell population leading to poor prognosis by a multivariate Cox proportional hazards regression analysis using at most three covariates of age, gender, and cancer stage. In this analysis, samples in a certain cluster were used as references for each cancer type. A cluster resulting in hazard ratio (HR) >1 to all the other clusters was selected as a reference cluster (Supplementary Table 4). We found that at least one cluster was associated with the prognosis for seven of the 16 cancer types (BLCA, LGG, LIHC, LUAD, LUSC, SKCM, and UCEC) (Figure 5). Samples in cluster 1 in LIHC were associated with worse prognosis compared to those in cluster 3 (*P* = 0.002). Samples in clusters 1 and 2 in LUAD were associated with worse prognosis compared to those in cluster 5 (*P* = 0.034 and *P* = 0.024, respectively). This suggested more mutations are associated with worse prognosis in these cancer types. Contrarily, samples in cluster 3 in BLCA were associated with worse prognosis compared with samples in cluster 1 (*P* = 0.020). Samples in cluster 3 and 5 in UCEC were associated to worse prognosis compared with samples in cluster 1 (*P* = 0.049 and *P* = 0.045, respectively). These results indicate that accumulation of a large number of mutations is not necessarily associated with worse prognosis. Cancer cells occupied by a lower number of mutations occurring early in cancer development might be associated with worse prognosis in BLCA and UCEC. Thus, samples were associated with poor prognosis when fewer mutations occurred at carcinogenesis and survived during cancer development. This result indicated genomic instability is a trade-off between cost and benefit [12].

Worse prognosis was observed in samples in clusters 3 and 4 in LGG compared to those in cluster 5 (*P* = 0.023 and *P* = 0.010, respectively). Therefore, samples in LGG with higher m_Peak and lower m_MATH were associated with poor prognosis. Since frequencies of MF mutations in *IDH1*, which is one of the driver genes in LGG, were higher among samples in clusters 3, 4, and 5 (69.5%, 54.5%, and 59.5%, respectively), it was expected that other factors that increase the number of mutations from the early to mid-stage of cancer development may affect patient prognosis. We could not, however, identify any genes specifically mutated in samples in clusters 3 and 4.

The effects of VAF distribution in LUSC and SKCM on prognosis were more complicated than those in the five cancer types described above. Samples in cluster 4 in LUSC were associated with worse prognosis compared to those in cluster 1 (*P* < 0.001). Samples in clusters 3 and 4 in SKCM were associated with worse prognosis than those in cluster 2 (*P* < 0.001 and *P* = 0.001, respectively). In these cancer types, subclonal progression was presumed to be associated with poor prognosis.

### Classification of samples using the decision tree model

More accurate prognostic prediction can be performed using the three parameters than using one-dimensional measures. To generate simple splitting criteria for classifying samples into one of the five clusters of VAF distributions, we performed decision tree analysis (CART). First, we constructed a complex decision tree and then pruned the branches using complexity parameter (CP) = 0.1 [31]. We additionally used a 10-fold cross-validation analysis to test the accuracy of the algorithm in classifying samples into one of the five clusters.

**Figure 6:**
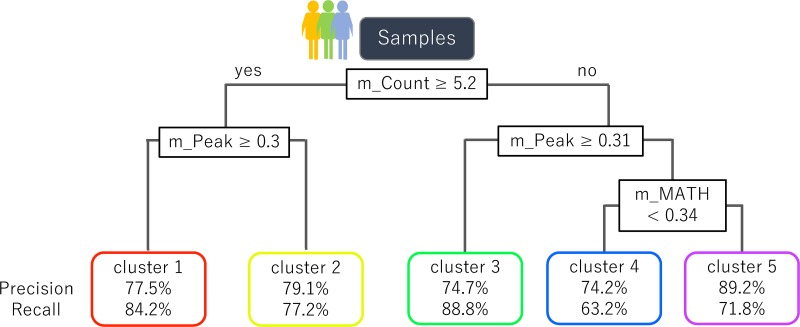
A decision tree to classify samples into the five clusters of VAF distributions A decision tree constructed using the three parameters derived from MF mutations is shown. The formula in the decision tree split criteria for samples. The nodes at the bottom of the tree indicate the five clusters and the corresponding accuracy of classification.

We created a decision tree with the three parameters used in this study. The maximum accuracy achieved by the classifier was 80.7%, and the average accuracy (±SD) was 76.8% (±2.0%), indicating that the decision tree model calculated from the three parameters can be used to a certain extent for classifying samples into one of the five clusters (Figure 6).

## DISCUSSION

Higher ITH has been implicated in poor cancer prognosis [1, 32–34]. Previous studies have used one-dimensional measures of ITH to analyze the relationship between ITH and clinical outcomes [13, 14, 35]. However, Andor and colleagues demonstrated a nonlinear association between the number of clones in tumor cells and prognosis [12]; thus, it is necessary to evaluate cancer heterogeneity from multidimensional viewpoints. To evaluate ITH multidimensionally, we used three kinds of parameters defining the shape of VAF distributions of each sample to divide those samples into five clusters. The shape of VAF distribution of each cluster had distinct genetic and genomic characteristics and were used to infer the evolutionary pathway of cancer cells. Moreover, we performed survival analyses for samples in each cluster and found that certain clusters were significantly associated with prognosis in seven cancer types. This result demonstrated it is possible to predict the preferable cancer cell status during evolution using VAF distribution. We also found that VAF distribution associated with worse prognosis varied considerably among cancer types. Although cancer cells are generally thought to accumulate multiple mutations during cancer development, our results showed that a larger amount of mutations are not necessarily associated with worse prognosis.

For SKCM samples, we obtained remarkable results from VAF distribution analysis. Previous studies have shown that melanoma is a highly malignant cancer and harbors various mutations in the early stages of cancer development [17, 36–38]. Melanoma is a highly aggressive cancer that tends to metastasize to various body tissues, leading to drug resistance via changing clonal composition [12, 39]. Our results consistently showed that most samples have a large number of mutations accumulated prior to carcinogenesis. The samples, which had fewer mutations and a branched evolutionary pattern, yielded worse prognosis than samples with a larger number of mutations. Taking the mutation spectrum into consideration, most mutations in the samples with fewer mutations were considered to occur in the later rather than earlier stages of cancer development. The proportion of samples in clusters 2 and 4 was significantly higher in the higher rather than the lower tumor stage. From these results, we proposed the following hypothesis of the genetic evolution of melanoma: melanoma is generated by a large number of genetic mutations, including those in *BRAF* (clusters 1 and 2) [38], and only those cells with certain mutations are selected under selective pressure. Highly malignant cancer cells with fewer mutations are then occupied in the cancer cell population. Those cells are possibly associated with a poor prognosis (clusters 3 and 4). Other mutated genes may be involved in evolutionary process of melanoma because of the low frequency of driver gene mutations in samples with few mutations.

As with melanoma, a large number of clonal mutations is known to occur in non-small cell lung carcinoma (NSCLC) [6]. Recently, tumor mutation burden (TMB) is used as a biomarker to assess response to immune checkpoint inhibitors in NSCLC treatment [40]. Previous studies showed that high TMB in NSCLC was associated with worse prognosis [41]. In LUAD, the study results were consistent with our finding that samples with more mutations have a poor prognosis (clusters 1 and 2). Therefore, the number, not the timing, of mutations might have a greater effect on the prognosis. In LUAD, the mutations partially attributable to smoking may gradually accumulate in cells during cancer progression, leading to more aggressive cancer cells. Conversely, in LUSC, once mutations are occupied in cancer cells under selective pressure (cluster 4), those samples were predicted to have a worse prognosis than cancer cells with a large number of clonal mutations (cluster 1). These cell populations might promote tumor growth and metastasis.

Even using three parameters separately, we could predict the prognosis of cancer patients. However, it will be difficult to infer the evolutionary process of cancer cells via a single variable. For example, even if there is an association between the number of mutations and prognosis, it will be difficult to infer the timing of such mutations. Via multidimensional analysis, we shed light on the association between prognosis and the state of cancer evolution. Since our analysis made it possible to predict this relationship more accurately and easily, we can apply such methodology to prognosis prediction and effective treatments.

In this study, we analyzed 16 cancer types using only single nucleotide substitutions in genes. To understand the evolutionary process of cancer in more detail, we should analyze each cancer type independently using other types of mutations, such as indels and CNVs. By considering the mutation signatures and gene expression patterns, we will be able to obtain further information on cancer cells’ evolution and its impact on patient prognosis. Furthermore, if we can identify genetic characteristics that show a higher correlation with certain shapes of VAF distributions, they can be used as prognostic predictors or diagnostic markers. These characteristics include, for example, mutated genes, mutation accumulation in regulatory regions, changes in epigenetic modifications, and gene expression.

## MATERIALS AND METHODS

### Datasets

Somatic mutation data, which were identified by applying the Mutect2 software package to matched tumor-normal pairs, were downloaded from the TCGA data portal (https://portal.gdc.cancer.gov/). The following 16 cancer types were analyzed: bladder urothelial carcinoma (BLCA), breast adenocarcinoma (BRCA), cervical squamous cell carcinoma and endocervical adenocarcinoma (CESC), colon adenocarcinoma (COAD), glioblastoma multiforme (GBM), head and neck squamous cell carcinoma (HNSC), kidney renal clear cell carcinoma (KIRC), lower-grade glioma (LGG), liver hepatocellular carcinoma (LIHC), lung adenocarcinoma (LUAD), lung squamous cell carcinoma (LUSC), ovarian serous carcinoma (OV), prostate adenocarcinoma (PRAD), skin cutaneous melanoma (SKCM), thyroid carcinoma (THCA), and uterine corpus endometrial carcinoma (UCEC). We also downloaded associated CNV data and clinical data [42].

### Mutation analysis

In this study, we considered only point mutations with a coverage depth of ≥ 20. Moreover, we extracted the mutations considered to be MF if they were classified as “probably damaging” or “possibly damaging” by PolyPhen-2 [43, 44]. The MF mutations of amino acid substitution may have an impact on protein structures and/or functions, suggesting their possible involvement in cancer development or progression. Copy number status was combined with these mutation data. Mutations with CNVs were excluded from this study. That is, we extracted mutations with segment mean values between −0.2 and 0.2, and the number of probes ≥ 10 [45]. For each mutation, VAFs were calculated as the proportion of the variant allele reads to the total reads at the mutation site. The VAFs were adjusted with tumor purity estimated by the ESTIMATE R package [46].

### Calculation of parameters that define the shape of VAF distribution shape

We only used samples with ≥ 2 MF mutations to calculate the three parameters that define the shape of VAF distributions: the corresponding VAF with the maximum value for probability density function of VAF distribution of MF mutations (m_Peak), log2[the total number of MF mutations] (m_Count) and MATH score for MF (m_MATH) [13]. The m_Peak indicates the peak position of the VAF distribution, the m_Count indicates the size for distribution, and the m_MATH indicates the variation of VAFs. We used these three parameters because they define overall shape of VAF distribution for MF.

### Statistical analysis

Statistical analysis was conducted using the R software, version 3.3.1 (R Project for Statistical Computing, Vienna, Austria), and JMP Pro, version 13.0 (SAS Institute Inc., Cary, NC, USA). A χ^2^ test or Fisher's exact test (when ≥ 1 cell had an expected frequency of ≤ 5 in any clinical group) was used to compare categorical variables. Survival analysis was performed using only clusters with sample sizes ≥ 10 in each cancer type. For the survival analysis, HRs with 95% confidence intervals (95% CIs) were calculated using a Cox proportional hazards regression analysis in the R survival package (version 2.41-3). The package was also used to evaluate the proportional-hazards assumption. To classify the samples into five clusters, k-medoids clustering with squared Euclidean distance metric was conducted using the cluster package (version 2.0.6) in R. For comparison of more than two groups, we performed ANOVA followed by Tukey's honest significant difference test. For comparison of genetic and clinical characteristics among 16 cancer types, *P*-values were adjusted by Benjamini-Hochberg correction. *P*-values were considered statistically significant at < 0.05 (^*^ *P* < 0.05, ^**^ *P* < 0.01, and ^***^ *P* < 0.001).

## SUPPLEMENTARY MATERIALS FIGURES AND TABLES




