# Supplementary material for: Pan-cancer analysis of intratumor heterogeneity associated with patient prognosis using multidimensional measures
Source: Oncotarget. 2018 Dec 28;9(102):37689–99. doi: 10.18632/oncotarget.26485 (PMC6340877; doi:10.18632/oncotarget.26485)
Supplement: Supplementary file 1 [file oncotarget-09-37689-s001.pdf]

Pan-cancer analysis of intratumor heterogeneity associated with patient prognosis using multidimensional measures

SUPPLEMENTARY MATERIALS

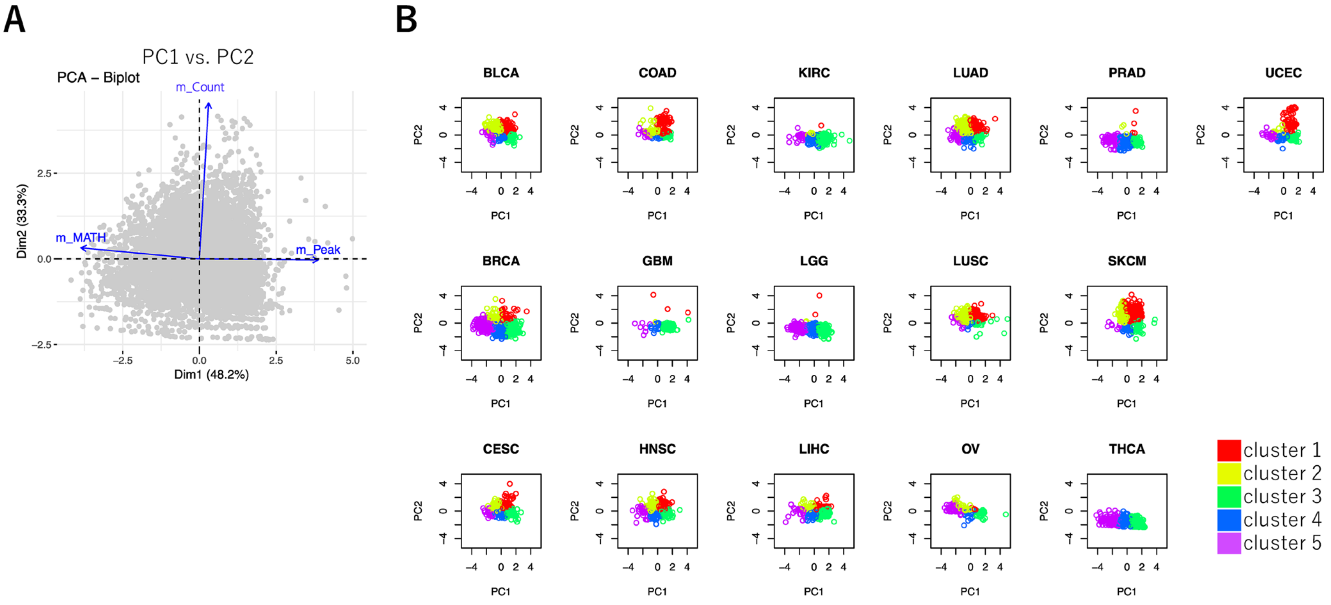

Supplementary Figure 1: PCA results using the three parameters derived from all samples in 16 cancer types. (A) Biplot represents PCA results using all cancer samples. Principal components, PC1 vs. PC2 is plotted here. Percentages of each axis indicate the contribution ratio, that is, the proportion of variance. **(B)** Scatter plots show samples in each cancer types divided into the five clusters. Color codes for the five clusters are indicated at the right side of the plots.

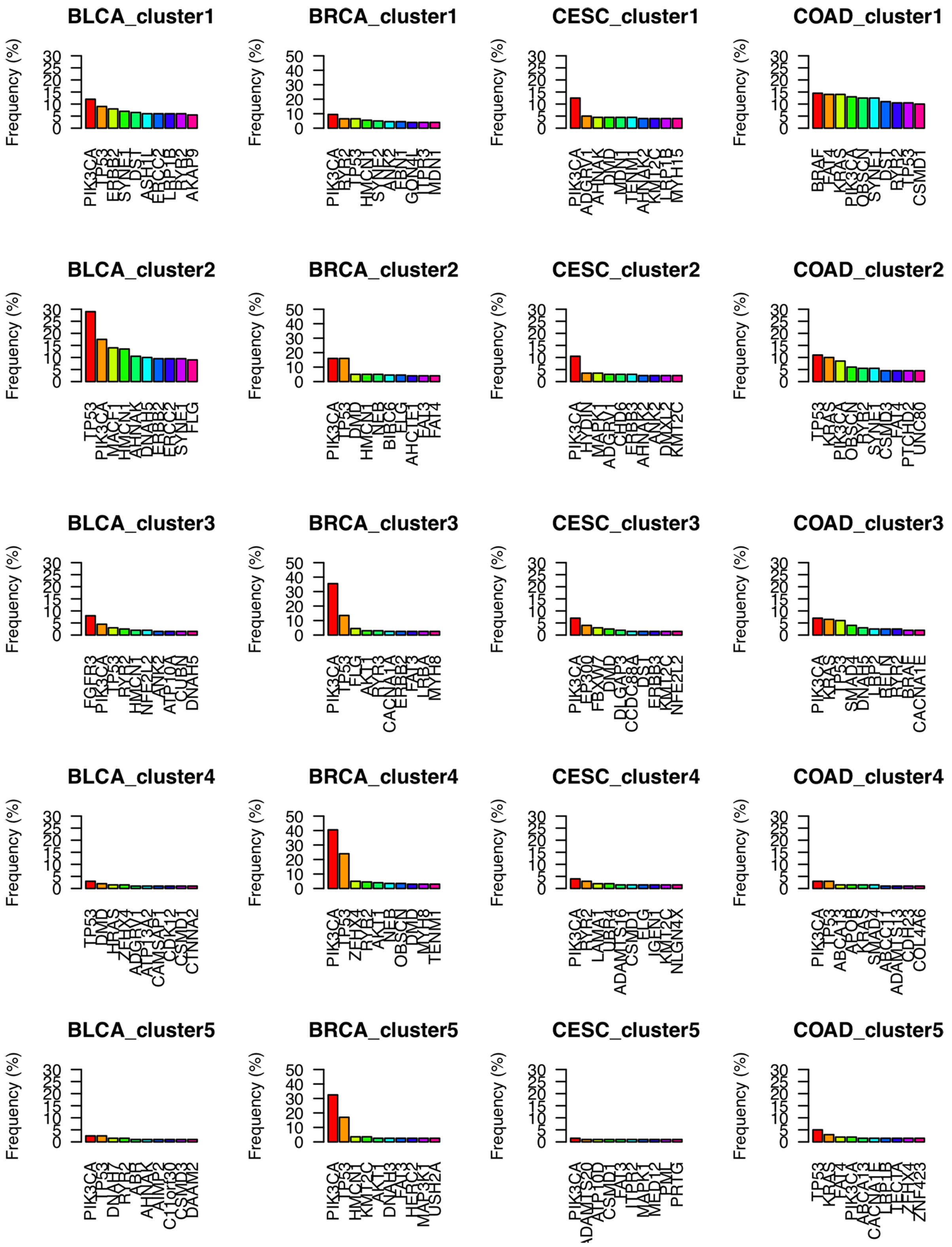

Supplementary Figure 2: MF mutation frequencies of each gene for 16 cancer types. Histograms represent MF mutation frequencies of genes for 16 cancer types. Ten genes with the highest frequency of mutations in each cluster are shown here. The vertical axis indicates the frequency of mutations.

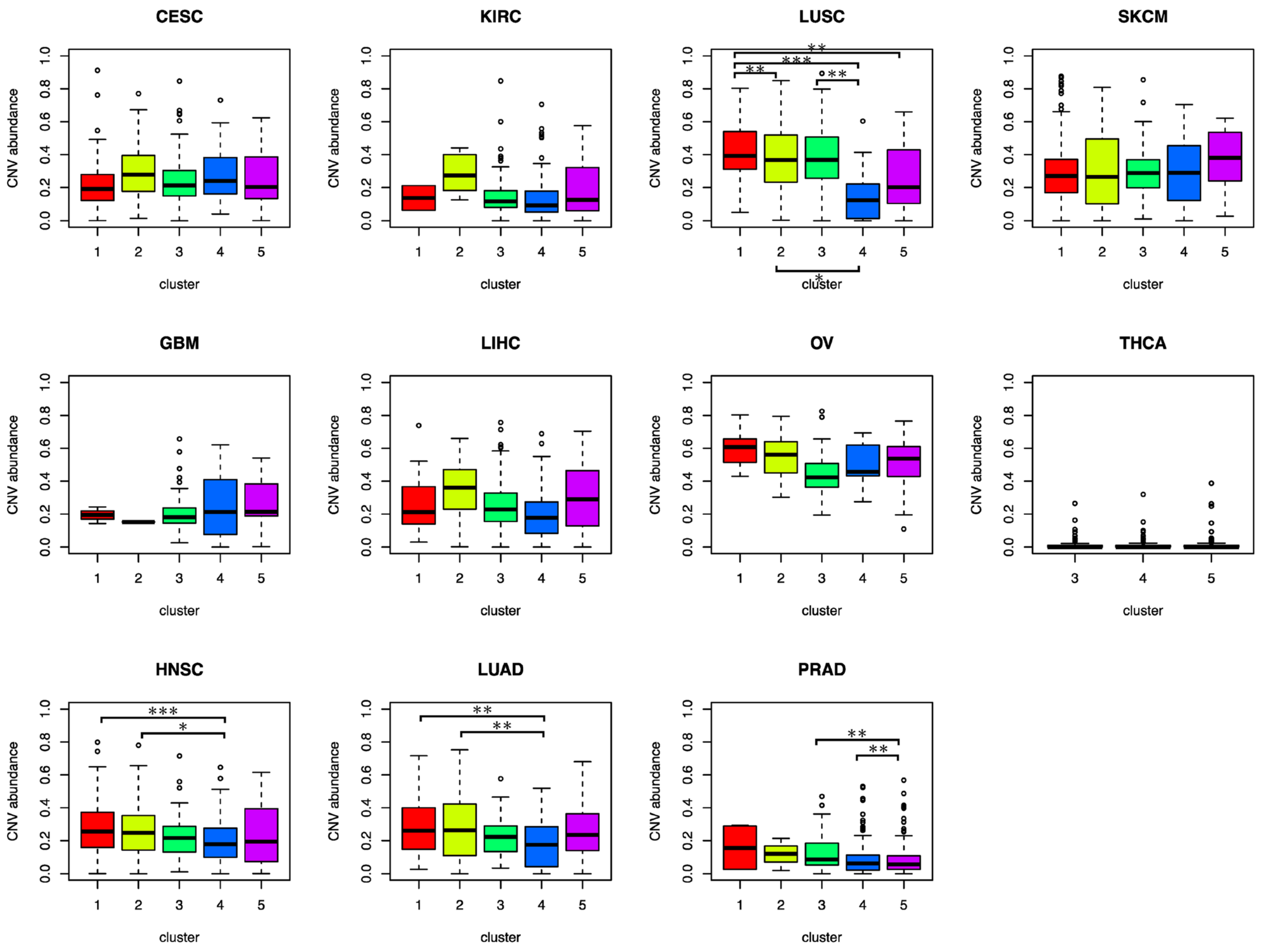

Supplementary Figure 3: Comparison of CNV abundance among the five clusters of VAF distributions. Boxplots for CNV abundance of the samples in each cluster are shown. ANOVA followed by Tukey's honest significant difference test was performed. $^{*}P < 0.05$, $^{**}P < 0.01$, and $^{***}P < 0.001$.

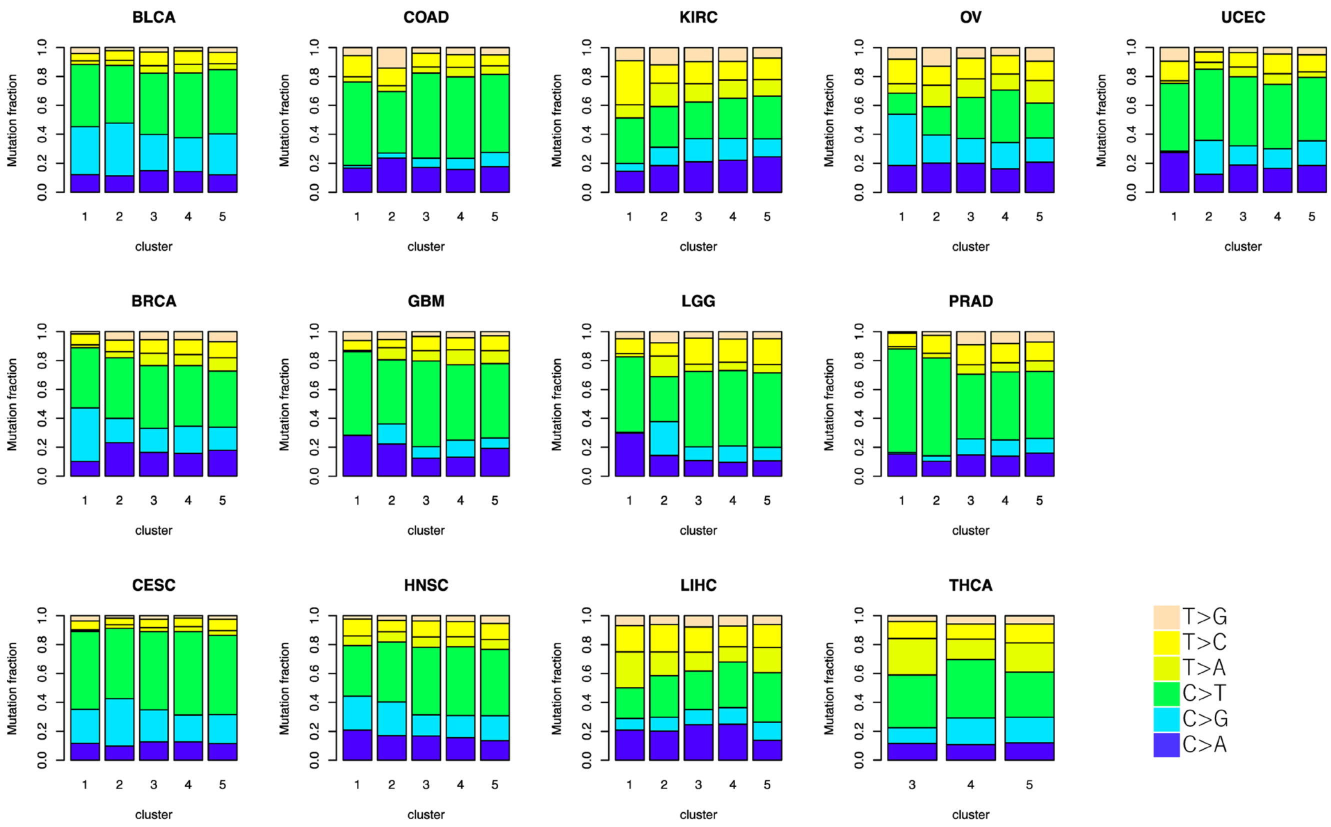

Supplementary Figure 4: Comparison of mutation spectra among the five clusters of VAF distributions. Bar plots for the frequencies of six mutation types in 13 cancer types are shown. The fractions of six mutation types in each cluster were shown.

Supplementary Table 1: Clinical characteristics and parameters of VAF distribution.

See Supplementary File 1

Supplementary Table 2: Clinical and genetic characteristics in each five cluster for 16 types of cancer.

See Supplementary File 1

Supplementary Table 3: Univariate Cox regression analysis of five clusters.

See Supplementary File 1

Supplementary Table 4: Multivariate Cox regression analysis of five clusters and clinical characteristics.

See Supplementary File 1
